# Supplementary material for: Soybean Yield and Nutrition Grown on the Straw of Grain Sorghum Inoculated with Azospirillum brasilense and Intercropped with BRS Paiaguás Grass
Source: Plants (Basel). 2023 May 17;12(10):2007. doi: 10.3390/plants12102007 (PMC10221422; doi:10.3390/plants12102007)
Supplement: Supplementary file 1 [file plants-12-02007-s001.zip › Supplementary Tables.pdf]

Supplementary Table S1. Photoperiod at Selvíria, Mato Grosso do Sul State, Brazil, during the study period.

Period	Month				
	November	December	January	February	March
	Photoperiod, h day^{-1}				
Monthly average for 2015/16 and 2016/17	13.2	13.3	13.2	12.8	12.2
Long-term (50-yr) average	13.1	13.4	13.2	12.8	12.2

Supplementary Table S2. Information on the cultivar, sowing date, spacing between rows, sowing density, fertilization, top dressing date and date of harvest or management for the species used in the cropping systems from 2015 to 2017.

Crop	Cultivar	Sowing date	Spacing between rows	Seeding density	Basic fertilization	Top dressing date	Top dressing	Date of harvest/ management
Sorghum (Off-season)	cv. Ranchero	17 Mar. 2015	0.45 m	10 seeds m^{-1}	[a]0 kg N ha^{-1}<br>[b]36 kg N ha^{-1}<br>[c]120 kg N ha^{-1}<br>40 kg P ha^{-1}<br>25 kg K ha^{-1}	24 Apr. 2015	[a]120 kg N ha^{-1}<br>[b]84 kg N ha^{-1},<br>[c]0 kg N ha^{-1},	18 June 2015
Sorghum (Off-season)	cv. Ranchero	06 Apr. 2016	0.45 m	10 seeds m^{-1}	[a]0 kg N ha^{-1}<br>[b]36 kg N ha^{-1}<br>[c]120 kg N ha^{-1}<br>40 kg P ha^{-1}<br>25 kg K ha^{-1}	13 May 2016	[a]120 kg N ha^{-1}<br>[b]84 kg N ha^{-1},<br>[c]0 kg N ha^{-1},	26 July 2016
Palisade grass (Off-season)	*U. brizantha* cv. BRS Paiaguás	17 Mar. 2015	Between the sorghum rows	10 kg ha^{-1} (60% seed viability).	-		-	18 June 2015
Palisade grass (Off-season)	*U. brizantha* cv. BRS Paiaguás	06 Apr. 2016	Between the sorghum rows	10 kg ha^{-1} (60% seed viability).	-		-	26 July 2016

Soybean (Season)	cv. BMX Potência RR	17 Nov. 2015	0.45 m	19 seeds m^{-1}	26 kg P ha^{-1}, 50 kg K ha^{-1}	-	17 Mar. 2016
Soybean (Season)	cv. BMX Potência RR	15 Nov. 2016	0.45 m	19 seeds m^{-1}	24 kg P ha^{-1}, 46 kg K ha^{-1}	-	03 Mar. 2017

[a] Basic fertilization: 0 kg N ha^{-1} - Top dressing: 120 kg N ha^{-1} (0% – 100%); [b] Basic fertilization: 36 kg N ha^{-1} - Top dressing: 84 kg N ha^{-1} (30% – 70%); [c] Basic fertilization: 120 kg N ha^{-1} - Top dressing: 0 kg N ha^{-1} (100% – 0%);
